# Isotopic imaging with epithermal neutrons at the ISIS Neutron and Muon Source

**DOI:** 10.1038/s41598-025-04283-y

**Published:** 2025-06-02

**Authors:** Giulia Marcucci, Antonella Scherillo, Davide Raspino, Daniela Di Martino

**Affiliations:** 1 STFC ISIS Neutron and Muon Source, Rutherford Appleton Laboratory, Didctot, UK; 2 Dipartimento di Fisica “G. Occhialini”, Università degli Studi di Milano-Bicocca and INFN Sezione di Milano-Bicocca, Milano, Italy

**Keywords:** Neutron imaging, Isotopic imaging, Epithermal neutrons, Nuclear resonances, Contrast enhancement, Silver isotopes, Experimental nuclear physics, Imaging techniques

## Abstract

An advanced non-invasive isotopic imaging technique based on Neutron Resonance Transmission Imaging (NRTI) has been implemented at the INES beamline of the ISIS Neutron Muon Source (UK), featuring high sensitivity to isotopes and enabling enhanced contrast in bulk radiography. NRTI relies on the neutron resonant absorption reactions occurring at epithermal energies. Nuclear resonance energies are isotope fingerprints, since the energy position of resonance dips in the transmitted neutron spectrum is uniquely associated with the individual isotopes present in the sample. Using a time and space-resolved detector with an event-mode acquisition system it is possible to conduct simultaneous spectroscopy and imaging investigation by recording the transmitted spectra in each pixel. Resonance selections can be performed post-processing through specific data analysis tools to enhance the absorption contrast of selected isotopes and visualise their distribution in the bulk. A pilot study with samples enriched with Ag-109 and Ag-107 is presented to demonstrate the potential of NRTI at the ISIS facility as a powerful and competitive tool for isotopic imaging. Future quantitative calibration for accurate isotopic ratio evaluation will expand its utility across various fields, such as nuclear engineering and archaeology, enabling detailed non-invasive analysis of complex materials previously challenging with conventional methods.

## Introduction

Energy-dependent neutron imaging has gained significant attention in recent years due to its unique capabilities in probing materials, providing a broad range of contrast mechanisms and becoming indispensable in modern research. Current advances in neutron imaging are driven by improvements in spatial resolution through detector technology research^1,2^, increased accessibility to high-flux neutron sources worldwide, and the integration of advanced computational techniques for data analysis, among which emerging artificial intelligence tools^3–5^. Developments in this field also crucially involved new contrast methods such as wavelength-selective imaging^6^, phase contrast^7^, grating interferometry^8^, polarization contrast imaging^9^, diffraction imaging^10^ etc., and wider applications for sensing various microstructural and compositional properties of materials that are otherwise difficult to study with conventional methods.

Updated reviews of the current state of neutron imaging can be found for example in^11–13^ and references therein, while detailed fundamental neutron properties and theoretical principles of neutron-matter interaction are given in^14,15^. Among neutron intrinsic properties that make them a suitable tool for imaging purposes, their penetrating power in dense material is invaluable for non-invasive bulk analysis. They are particularly effective for observing light atoms and distinguishing neighbouring elements in the periodic table thanks to different attenuation with respect to other probes like X-rays.

**Fig. 1:**
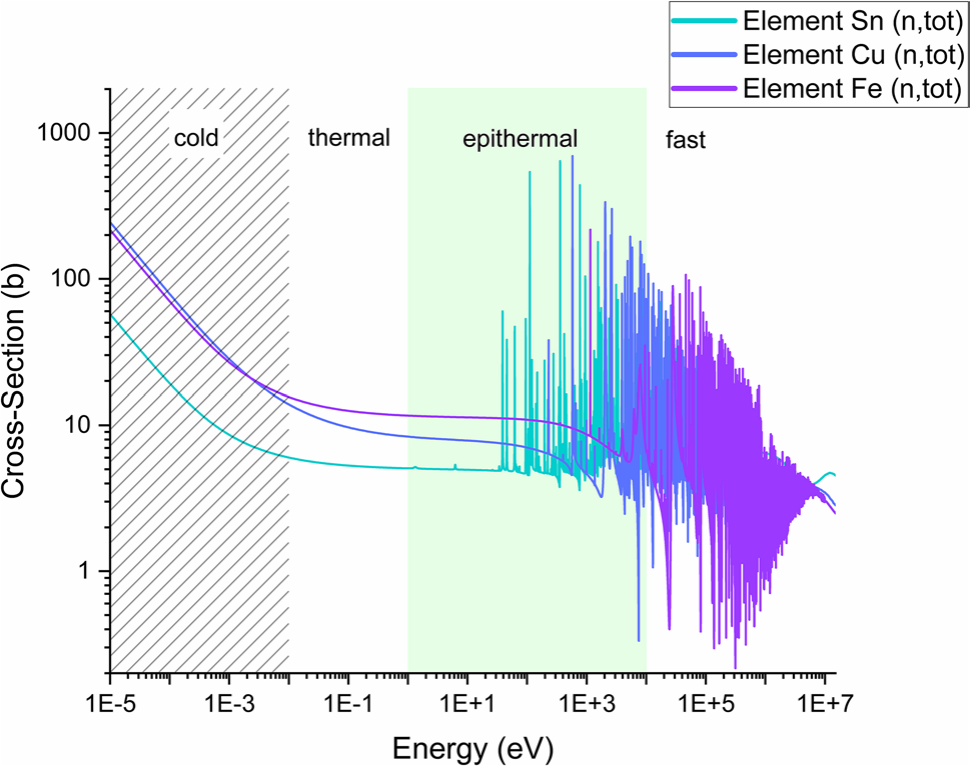
(n,tot) cross-section of elements Sn, Cu and Fe from the evaluated nuclear data library ENDF/B-VIII.0^19^. The plot illustrates cross-section values over different ranges of neutron energies: the dashed area highlights cold neutrons (energies below 0.01 eV); thermal energies are between 0.01 eV and 0.5 eV; the green area comprises epithermal energies (0.5 eV—10 keV); fast neutrons are above 10 keV. Conventional neutron imaging relies on the different attenuation powers of elements in the cold region, however some elements or composition materials could coincidentally have the same values, such as Fe and Cu. In the epithermal region, resonance structures are clearly elemental (and isotopic) fingerprints.

Neutrons can be classified by their energies. Within this article, we will use the following definitions. Cold neutrons: energies below 0.01 eV; thermal neutrons: energy range from 0.01 eV to 0.5 eV; epithermal neutrons: from 0.5 eV to 10 keV; fast neutrons: above 10 keV. Conventionally, neutron imaging mostly relies on cold neutron beams, with material identification determined by variations in attenuation coefficients (Fig. 1). However, composite materials could coincidentally have the same macroscopic attenuation properties for slow neutrons leading to ambiguous identification and achieving adequate contrast to differentiate between elements with similar attenuation power could be challenging.

At epithermal energies, pronounced resonance structures dominate the neutron absorption interaction with nuclei, and the energy positions of these peaks are unique to each nuclide. Notably, not only do different elements exhibit distinct resonances in terms of energy position, but isotopes of the same element are also characterised by their peculiar fingerprint: neutrons are the only scattering probe which can provide isotopic contrast. Currently, only a few sources worldwide provide epithermal neutron beams, typically among which are neutron spallation sources. Exploiting the epithermal neutrons, we are implementing the innovative Neutron Resonance Transmission Imaging (NRTI) technique at the ISIS Neutron and Muon Source (UK). At present, the NRTI technique is not readily available as an en-suite technique within large-scale facilities. However, different neutron sources are exploring this opportunity besides ISIS, such as the Los Alamos National Laboratory^16^ and the Spallation Neutron Source (SNS) in the US^17^, and the Japanese Proton Accelerator Research Complex (J-PARC) in Japan^18^.

NRTI is based on time-of-flight (ToF) measurement of the transmitted neutron beam passing through an object. The typical transmission curve presents negative dips whose positions in energy correspond to the absorption resonances responsible for the neutron removal from the incident beam. With adequate time resolution, the position of these dips allows for clear isotopic identification through comparison with the (n, tot) cross-sections of nuclides available in dedicated nuclear databases, such as^19^. According to nuclear physics notation, (n, tot) represents the total reaction cross-section between a nucleus and the neutron. This imaging method enables for distinguishing and mapping, with enhanced contrast, elements and isotopes within a sample that would be undetectable and/or indistinguishable using conventional imaging methods.

The NRTI optimisation specifically at ISIS has been an ongoing research effort since 2007, within the CNR-STFC collaboration and the Ancient Charm project^20^. The initial implementation focussed on the viability demonstration for two- and three-dimensional (2D/3D) mapping of elements using a low-resolution position-sensitive ToF detection system at the INES beamline, with the first proof of concept in a cultural heritage-related sample^21,22^. Subsequent improvements in the detection system (resolutions and efficiency) and data handling procedures (processing, normalisation, and toolkit for post-processing analysis) have led to the creation of a consolidated toolkit for 2D elemental mapping^23,24^. As a result, bulk elemental mapping with NRTI is now effectively available to the scientific community, enabling the submission of official beamtime requests within the user programme for conducting experiments at the INES beamline of ISIS.

Following previous implementations, we explored enhanced isotopic imaging as an application of NRTI with the aim of exploiting its unique features where mapping the distribution of isotopes is relevant. Compared to the few existing isotopic investigation techniques, such as mass spectrometry (ICP-MS) or thermal ionization mass spectrometry (TIMS), NRTI can offer complementary and spatially resolved bulk information in a non-destructive way and without the need for sample preparation. Previous research works have already demonstrated the potential of resonance absorption spectroscopy for the quantification of isotope densities within homogeneous samples with both two- and three-dimensional imaging measurements at pulsed neutron sources^25–28^.

The primary aim of this article is to emphasise the potential of the NRTI setup optimised at the ISIS facility for isotopic mapping with contrast enhancement in inhomogeneous samples by presenting a feasibility study on certified samples enriched with Ag isotopes (details on the sample compositions are provided in the Material and Methods section). Applications specifically focused on mapping the distribution of isotopes are not yet widespread, partially due to technological limitations. Interest has grown in certain areas such as nuclear engineering^16^ or cold fusion where, for example, silver is produced with an anomalous isotopic distribution during experiments involving the electrolysis of heavy water with palladium cathodes^29^. Further potential applications can be found in archaeology, since the estimation of silver but also other isotopic ratios are essential for tracing the origin of ancient artefacts. Silver isotopic ratio, in particular, is used to investigate historical trade routes and the origin of coinage coins^30–32^, but so far only destructive methods are available, and they are not always acceptable from a conservation point of view. At present, we propose this technique as a non-destructive imaging method to map the isotopes into materials. In addition, given the results obtained with Ag isotopes, we envisage to extend NRTI application to other isotopes which have similar attenuation coefficients to ^107^Ag and ^109^Ag, to expand the use of NRTI in mapping isotope distributions beyond silver.

## Results

### Isotopic resonance selection for contrast enhancement

**Fig. 2:**
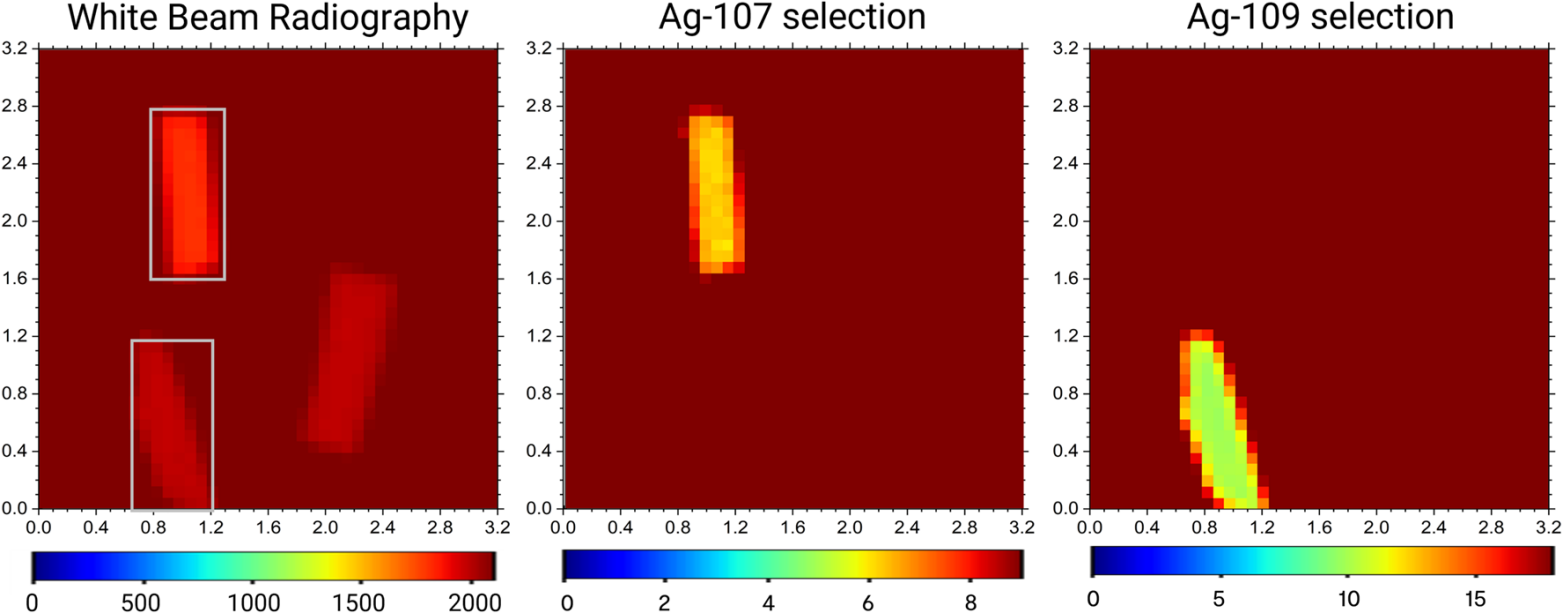
NRTI maps of silver flakes before and after contrast enhancement. Left: 2D white beam radiography of two silver flakes and one Pb sample acquired with NRTI. Each image pixel contains the full transmission spectrum in the 0–2 ms ToF range available at the INES beamline. Centre: NRTI map after the selection of the main resonance of ^107^Ag around 16.3 eV (Fig. 3). The selection highlights the sample enriched with 99.7% ^107^Ag, which emerges with enhanced contrast. Right: NRTI map after the selection of the main resonance of ^109^Ag around 30.6 eV (Fig. 3). In this case, the sample enriched with 99.7% ^109^Ag is enhanced. The size of the maps is 3.2 × 3.2 cm^2^, with 0.8 mm of pixel size. For all the images, the colour scale refers to the transmission intensity over the selected ToF range.

Two samples with different isotopic enrichment (Ag-enr-109 and Ag-enr-107, defined in section Materials and Methods) have been irradiated using the NRTI setup available on the INES instrument^23,24,33^ operating at the ISIS Neutron and Muon Source. Details about experimental setup and data acquisition/normalisation are given in section Materials and Methods. The normalised transmission radiography is displayed in Fig. 2: at this stage, the recognition of the isotope is not straightforward as the level of neutron absorption is similar between the two samples, apart from thickness variation.

**Fig. 3:**
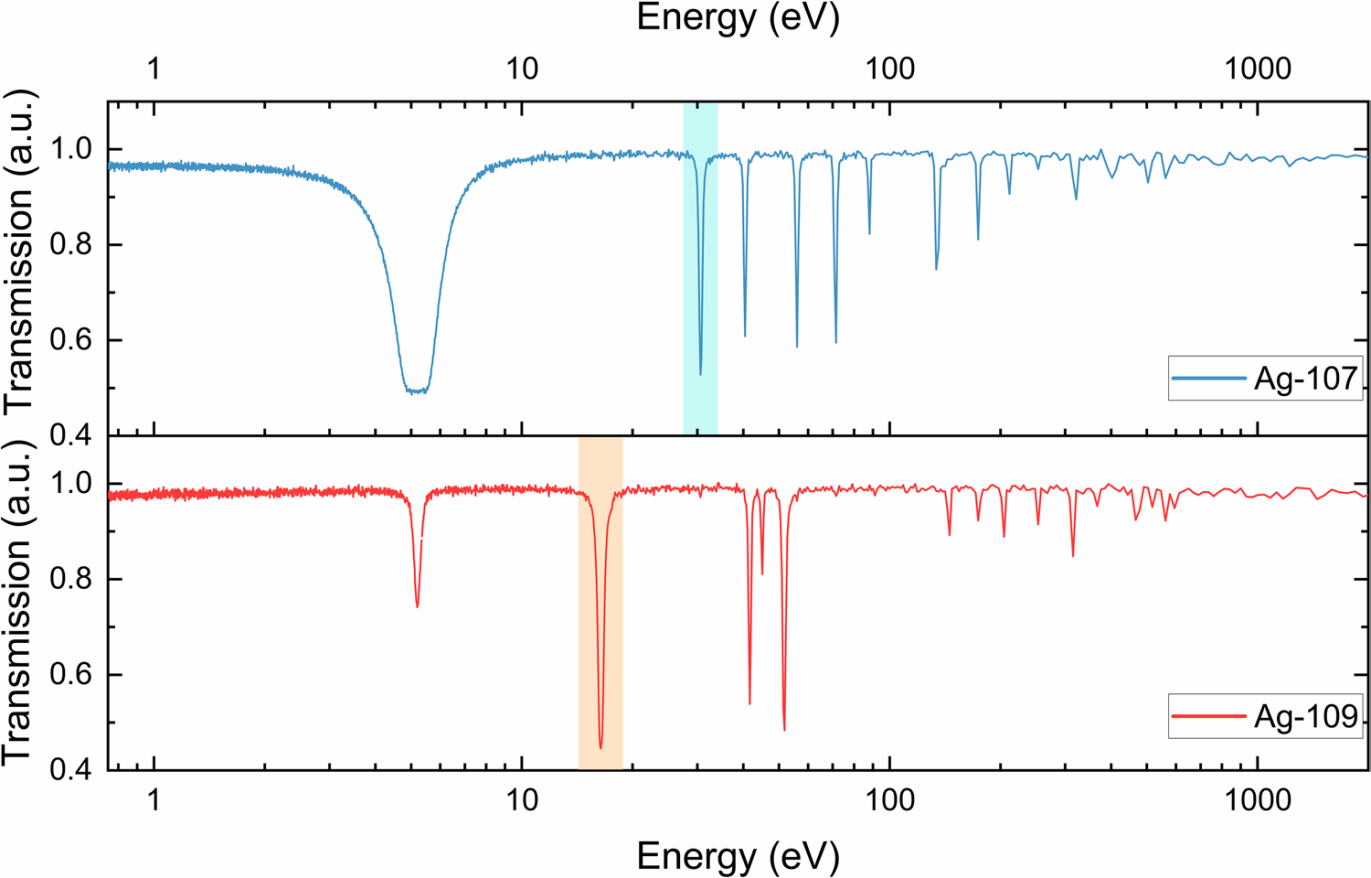
Transmission spectra corresponding to the selection of a ROI containing only one of the two enriched samples, respectively. The resonance positions in the blue spectrum clearly indicated the presence of ^107^Ag, while the ^109^Ag resonances are identified in the red spectrum, along with minor dips caused by the small amount of ^107^Ag still present in the sample. The light blue and orange boxes highlight the two energy regions used during the resonance selection process.

**Fig. 4:**
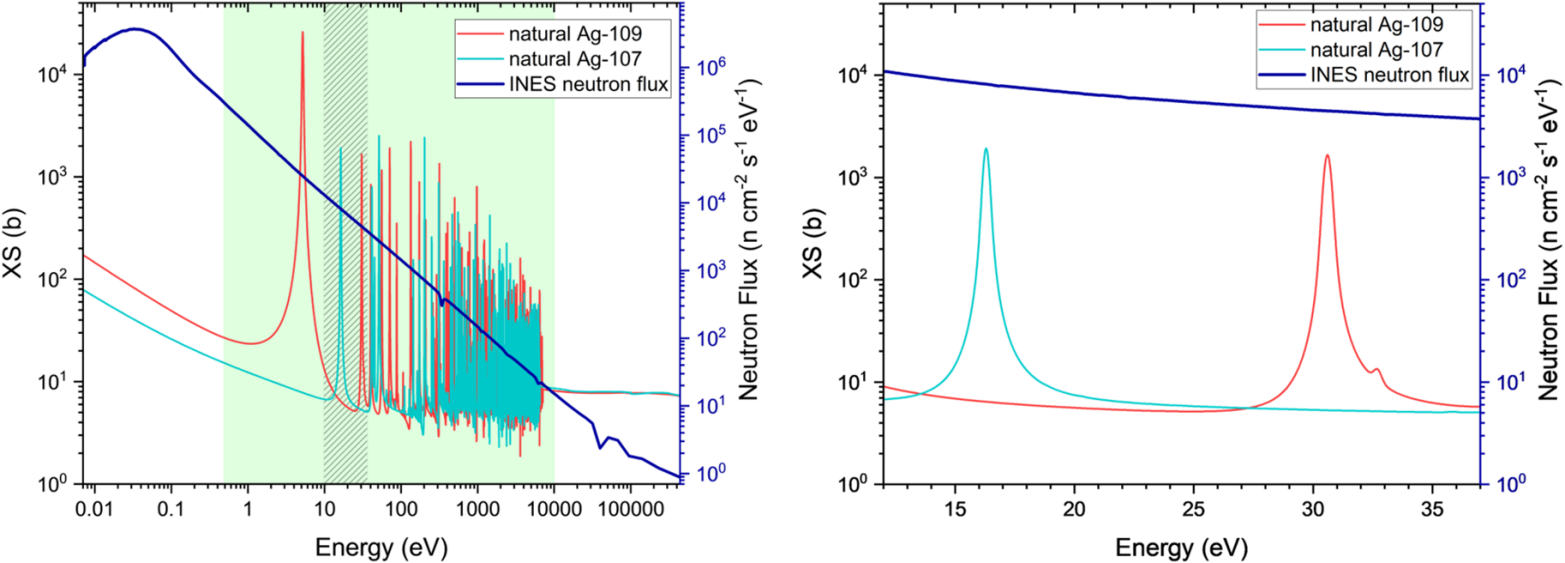
(n,tot) interaction cross-sections of the two stable isotopes of silver, ^107^Ag (light blue) and ^109^Ag (red). The cross-sections have been taken from the evaluated nuclear data library ENDF/B-VIII.0^19^. The neutron flux, experimentally measured at the INES beamline, is also displayed (thicker blue line). The green-shaded area delimited the epithermal energies. The dashed pattern highlights the energy region considered in this test study. On the left: an expanded view of the energy region containing the two resonances considered for contrast enhancement and isotopic identification.

For each sample, a region of interest (ROI) comprising its volume has been selected to extract the corresponding transmission spectrum, shown in Fig. 3. The isotopic composition of the samples was determined by comparing the position of the main resonance dips in the transmission spectra with the resonance peaks obtained from tabulated total neutron cross-sections (Fig. 4). The two silver isotopes are characterised by two intense resonances falling in well-separated energy positions (^107^Ag @ 16.3 eV, ^109^Ag @ 30.6 eV), which allow them to be distinguished from each other through the analysis of transmission spectra giving the present energy resolution of the NRTI experimental setup.

Furthermore, within the Mantid application^34,35^ it is possible to narrow the ToF - or, equivalently, the energy- range to be visualised in the 2D map. In this way, a ToF/energy range comprising only one resonance can be selected after collecting the data in event mode, enhancing the absorption signal due to the energy selection over the entire image. The resonance selection process is performed post-irradiation, after data normalisation, through specific tools implemented in the Mantid project, an open-source software created to manipulate and analyse neutron scattering and muon spectroscopy data (but could be applied to many other techniques). Further details on the resonance selection process can be found in the Supplementary Material.

As shown in Fig. 2, the selection of the neutron energies pertaining to the resonance related to only the ^107^Ag (light-blue mask in Fig. 3) allowed to visualise the distribution of this isotope in the samples and therefore to distinguish which flake is mainly composed of ^107^Ag with respect to ^109^Ag. Similarly, the selection of neutron energies pertaining to the ^109^Ag resonance (orange mask in Fig. 3) enhances the localization in the radiography of the sample enriched with 99.70% of this isotope. The most intense resonance of ^107^Ag falling around 5.2 eV can not be used for this scope (contrast enhancement) since its 0.3% contamination in Ag-enr-109 flakes still causes a resonant dip at this energy position.

### Isotopic mapping – comparison with conventional neutron imaging

**Fig. 5:**
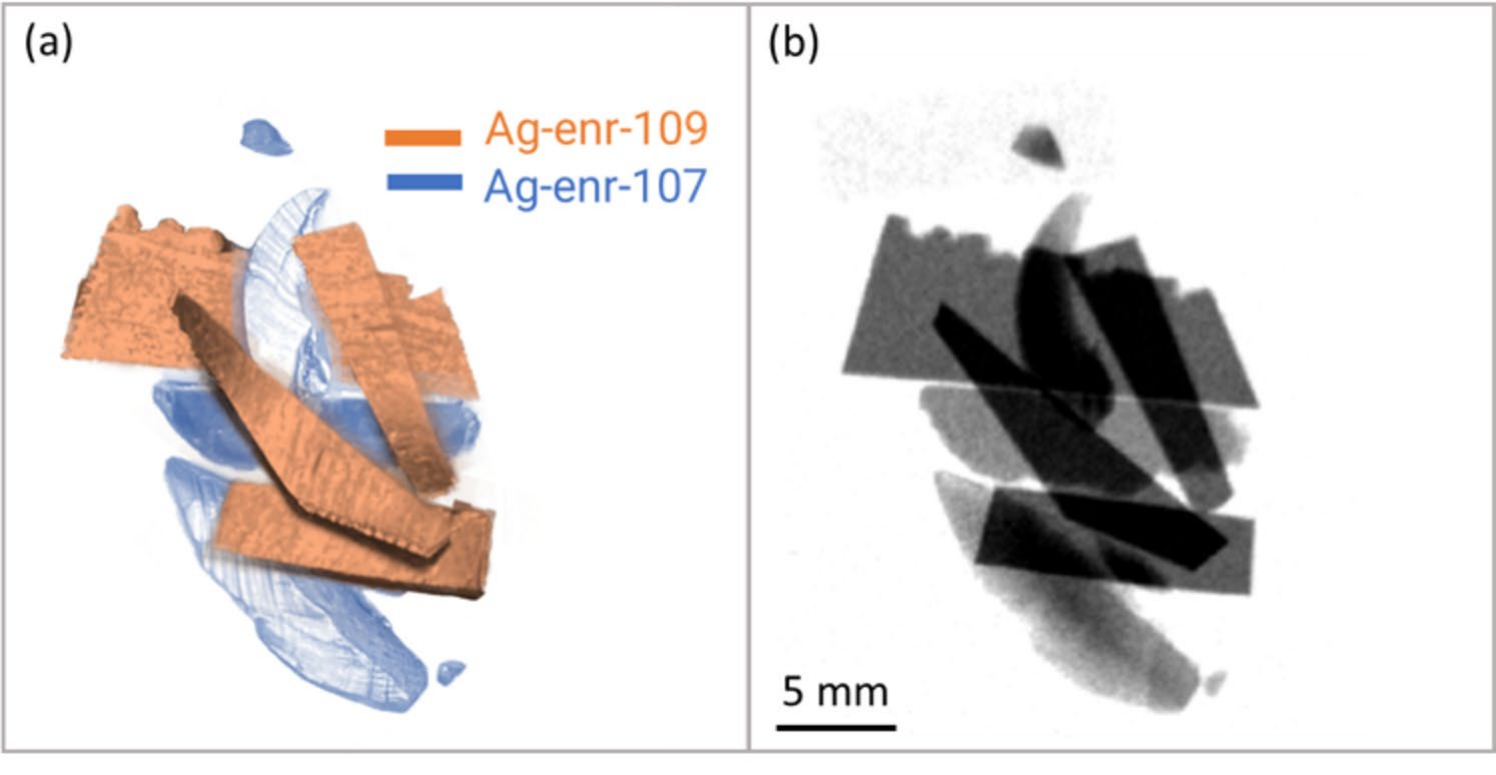
(**a**) False-colour image of the sample composed of different flakes with varying enrichment levels: Ag-enr-109 samples enriched with > 99% of ^109^Ag are highlighted in orange; Ag-enr-107 samples enriched with > 99% of ^107^Ag are highlighted in blue. (**b**) Standard cold neutron radiograph acquired at the IMAT beamline. The pixel size of the IMAT neutron camera is 0.048 µm.

**Fig. 6:**
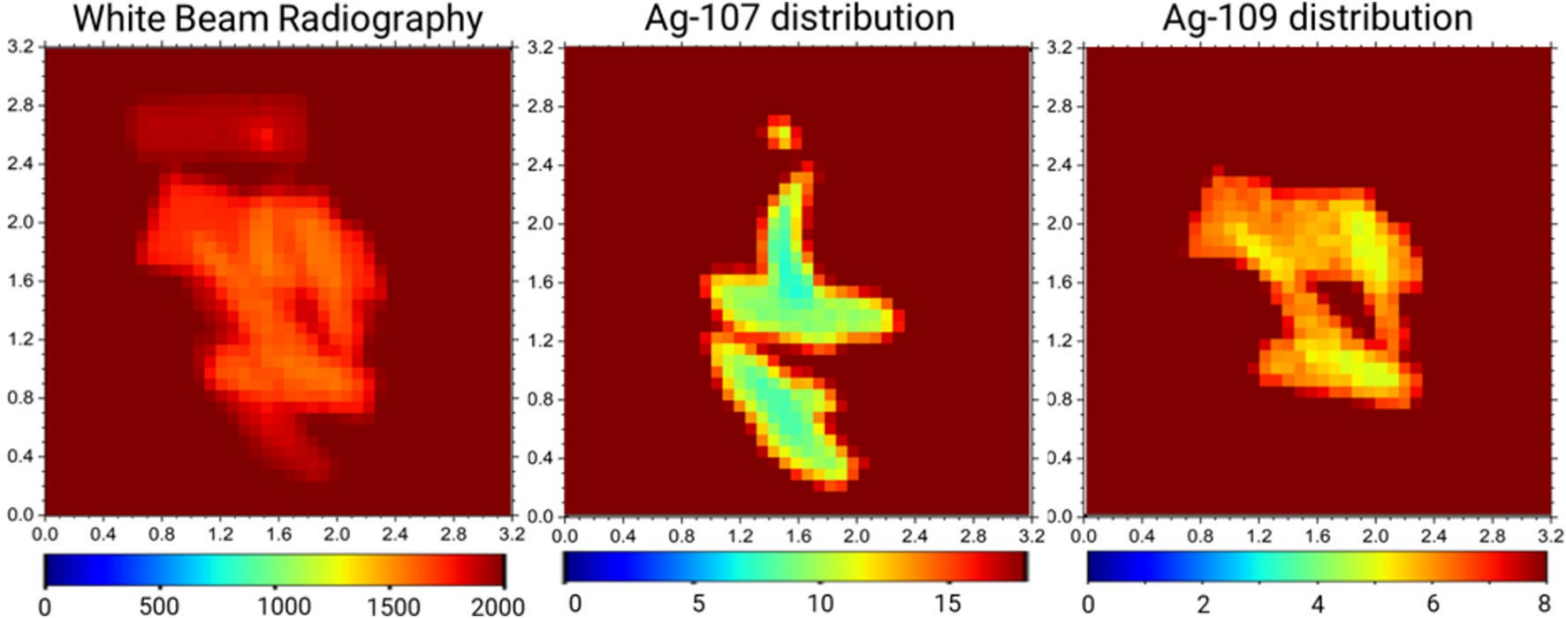
Transmission maps showing isotopic selections of ^107^Ag and ^109^Ag, alongside the white beam radiography for comparison, for the random aggregation of enriched silver flakes. Each image highlights the material distribution based on transmission intensities (T, in arbitrary units). The colour scales indicate the relative abundance of each isotope across the sample, with contrast variations revealing distinct elemental patterns and enrichment regions. The size of the maps is 3.2 × 3.2 cm^2^, with 0.8 mm of pixel size. For all the images, the colour scale refers to the transmission intensity over the selected ToF range.

**Fig. 7:**
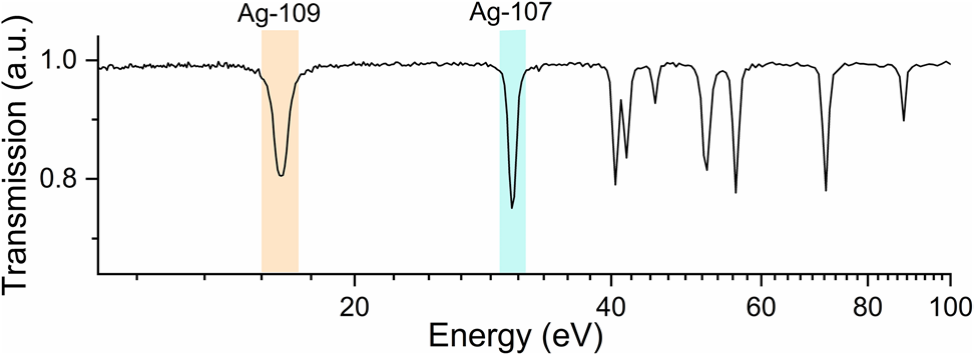
Close-up of the transmission spectrum of the random aggregation of enriched silver flakes in the energy range of interest for silver isotopes mapping (10–100 eV). In this case, the transmission is a combination of the two isotopic cross-sections displayed in Fig. 2. Light blue and orange boxes highlight the resonance peaks identifying the specific isotopes present in the sample and exploited for contrast enhancement in Fig. 6.

A random aggregation of the two types of enriched flakes has been produced with the scope to emphasise further the contrast enhancement for isotopic identification through NRTI and reproduce a more inhomogeneous structure closer to a real sample. The aggregation is shown in Fig. 5a, where the false colour can guide the isotope identification in the picture. In this case, standard radiography with cold neutron has been also acquired at the IMAT beamline^36^ of the ISIS facility, to compare the isotopic sensitivity of the two imaging methods (Fig. 5b). Through standard neutron imaging, only thickness effects are visible: the different grey level refers to the overlapping of two or more flakes or thickness inhomogeneity within the same flakes, while, as expected, it is not possible to distinguish between the two Ag isotopes. On the other hand, selecting one resonance specific for ^107^Ag or ^109^Ag allows the identification of the two kinds of enriched flakes leveraging the contrast which results in being amplified, as displayed in Fig. 6. In addition, attenuations due to overlying material are suppressed during resonance selections. The withe beam radiography in Fig. 6 shows similar information as the standard cold neutron radiography, *i.e.* different transmission levels are mainly due to variations in thickness. The main resonances of ^107^Ag and ^109^Ag have been identified from the transmission spectrum (Fig. 7) obtained by selecting the entire volume of the flakes aggregation acquired by NRTI.

## Discussion

The development of NRTI as an isotopic neutron imaging technique at the ISIS Neutron and Muon Source (UK) significantly broadens the insights of energy-selective imaging methods. While conventional neutron imaging based on cold neutrons already provides valuable information on material composition and structure, the isotopic sensitivity enables the mapping of isotopic distribution within the bulk of a sample exploiting the resonance structures present at epithermal energies available at spallation sources.

The method allows for the differentiation of isotopes, otherwise chemically identical for other elemental analyses based on more standard probes. Event-mode acquisition of ToF radiographies over the white neutron beam crucially differentiates this method from other imaging and isotopic techniques. This acquisition system enables simultaneous detection of neutron transmission spectra in function of the ToF at each detector pixel. During post-processing analysis, specific neutron energy ranges can be selected, corresponding to resonant dips occurring in the transmitted spectrum. The possibility of performing this selection step, called resonance selection, is particularly advantageous for investigating the elemental and isotopic composition and distribution of unknown materials. Moreover, through the resonance selection, the distribution of specific isotopes can be mapped in 2D with enhanced contrast, even though the isotopes have similar attenuation power for cold neutrons.

The pilot study described in this paper focuses on mapping of the two silver isotopes, ^107^Ag and ^109^Ag, which have reasonably high resonant cross-sections at epithermal energies for relatively fast measurements. However, the method is not limited to these nuclides but can be extended to the detection of isotopes with comparable or higher resonance (such as antimony, indium, etc.). On the other hand, for smaller cross sections this method can be applied considering two key factors beyond extending measurement times: the reaction rate (evaluated based on the resonance energy and the corresponding neutron flux at the same energy), as well as the amount of material.

Considering the ISIS proton beam double-pulse structure and the extension of the water moderator feeding the INES beamline, the energy resolution ΔE/E of the current imaging setup at INES has been estimated following^37^. In particular, ΔE/E varies from $4\cdot {10}^{-4}$ at 1 eV to $4\cdot {10}^{-2}$ at 10 keV: nuclear resonances at low epithermal energies are better resolved, allowing for improved differentiation of nuclides with closely spaced resonances within this resolution limit.

Isotopic NRTI can be also implemented for full tomography measurements; however, improvements to the setup efficiency are necessary to reduce scanning times. Currently, a single radiograph takes tens of minutes to complete with adequate statistical quality, given an average proton current of 130 µA delivered by the ISIS Neutron and Muon Source. Performing 3D enhanced isotopic imaging will make NRTI a powerful and more competitive method for routine use in tomographic applications as well as for material characterisations with a multi-technique approach.

Finally, a further step in the NRTI development will be its quantitative calibration to evaluate isotopic ratios in a completely non-invasive way. From this perspective, the broad energy range detectable at INES and the white neutron beam provided by the source may be beneficial if the main resonance of a nuclide is saturated, making it impossible to extract quantitative information from it (such as the Ag-107 resonance around 5.2 eV in Fig. 2). In such cases, secondary resonances - if they are available and not overlapping with those of other isotopes - can be considered instead during post-processing analysis, without the need to tune the neutron beam.

This advancement opens new perspectives for the research carried out at ISIS in a wide range of fields, such as materials science, nuclear engineering, superconductivity, and archaeology, allowing for more detailed analysis of complex materials, where the isotopic distributions were previously difficult to discern using conventional methods.

## Materials and methods

### Materials

Silver has two naturally occurring isotopes with similar abundance, ^107^Ag (51.84%) and ^109^Ag (48.16%). Silver samples in flakes with different isotopic enrichment from the brand CK Isotopes^38^ have been used for this study. The flakes are distinguished between the ‘Ag-enr-109’ type and ‘Ag-enr-107’ type (‘enr’ means ‘enriched’), which correspond to two kinds of enrichment. Ag-enr-109 flakes contain 99.7% of ^109^Ag, while Ag-enr-107 flakes are enriched with 99.5% of ^107^Ag. A detailed list of certified chemical impurities is available in Supplementary Table 1. Flakes geometry and dimension are displayed in Supplementary Fig. 4. The average thickness of the Ag-enr-109 and Ag-enr-107 flakes are 0.69 mm (with 0.005 mm^2^ of variance of thickness distribution) and 1.29 mm (0.06 mm^2^ of variance), respectively.

### NRTI setup

NRTI imaging has been developed at the INES beamline of the ISIS Neutron and Muon Source. The INES beamline received neutrons moderated with water at ambient temperature, resulting in a white neutron spectrum with a significant epithermal component (Supplementary Fig. 5).

**Fig. 8:**
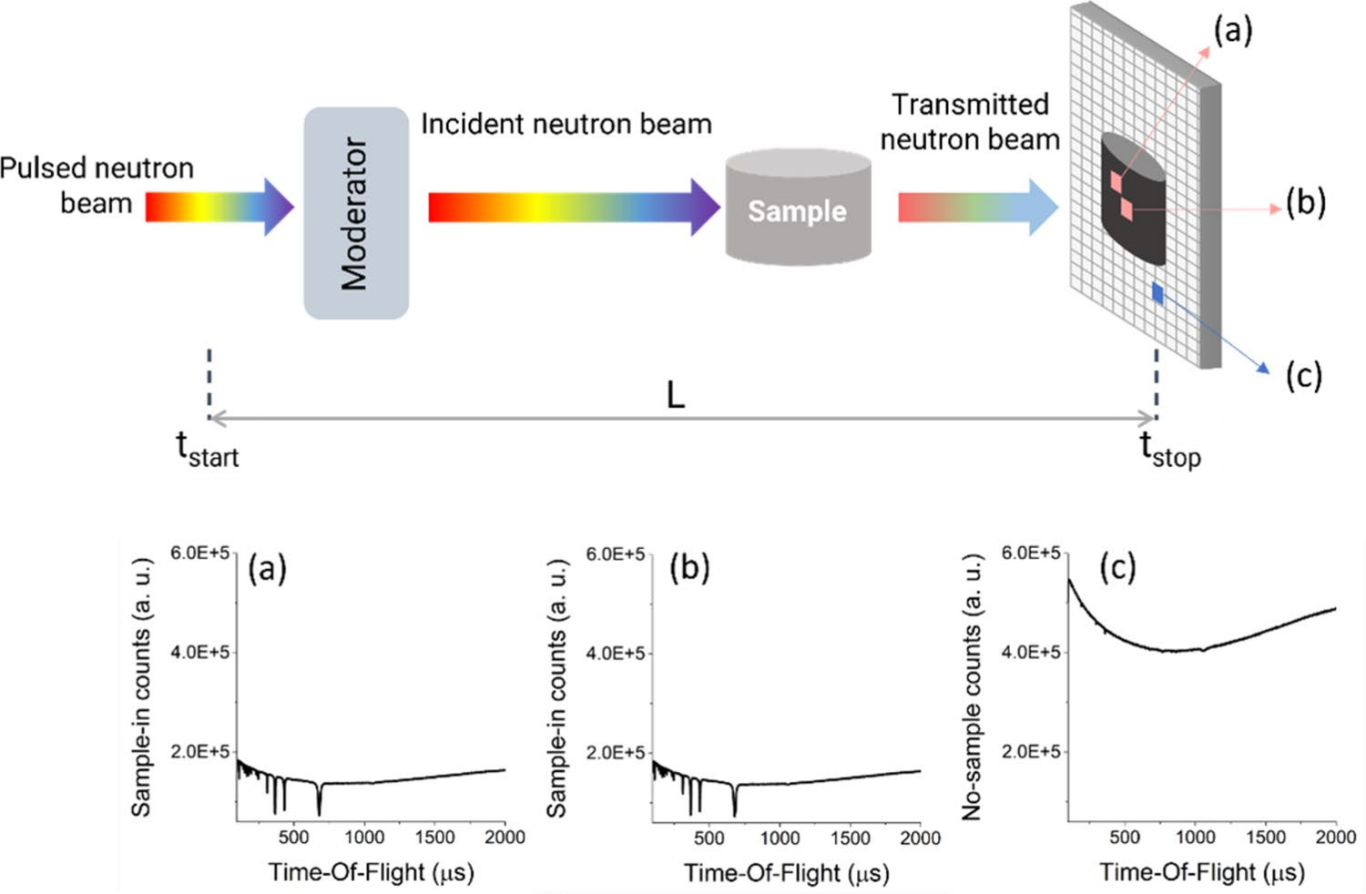
Schematic representation of the imaging setup for NRTI measurements at the INES beamline. The pulsed neutron beam is moderated through water at ambient temperature and then transmitted through the sample under investigation. Transmitted neutrons are detected by the position-sensitive nGEM detector. (**a**) and (**b**) show sample-in ToF spectra with characteristic resonant dips related to the sample composition extracted from different pixels, while (**c**) presents an open beam (or sample-out) ToF spectrum for reference.

A schematic layout of the NRTI experimental set-up is represented in Fig. 8. A commercial neutron Gas Electron Multiplier (nGEM)^39,40^ is currently employed and placed after the sample position to measure the transmitted neutron spectrum through the material, at a 23.44 m distance from the water moderator. For imaging purposes, samples are positioned as close as possible to the detector to reduce scattering events and blurring effects due to the beam divergence.

The active area of the nGEM detector is 10×10 cm^2^, with a pixel size of 0.8 mm. However, the effective area of investigation is limited to the maximum opening size of a jaws system (beam ‘delimiters’ made of neutron absorbing materials) present on INES, which is 4.0×4.0 cm^2^, and usually used to reduce the beam transverse section for diffraction measurements on small samples. The commercial detector can operate up to a total count rate of 10 MHz on the whole detector area. Moreover, each neutron is time-stamped with a precision of 8 ns referred to the T-zero of the ISIS accelerator (*i.e.* when the proton beam hits the target). Considering the time broadening of ∼400 ns affecting the initial neutron pulse due to the neutrons slowing down in the small water moderator^41,42^, the time binning of the NRTI acquisition has been set at 1 μs, but can be more resolved depending on the amount of data to be generated.

The pixelated detector operates in event-mode. The transmitted neutron beam is recorded in each detector pixel as a function of the neutron time-of-flight. This measurement is directly related to the theoretical transmission T, which represents the fraction of the neutron beam that passes through the sample without any interaction. Data acquisition relies on the event-mode system: each detection event is simultaneously recorded with a time stamp (neutron ToF) and (X,Y) coordinate of the pixel. With a total of 128 x 128 pixels, this method generates 16384 histogrammed data with ToF transmission spectra. For each spectrum, ToF, Y (counts) and E (error) data are stored. The advantage of event-mode acquisition is the possibility of extracting signals at specific neutron energies after the measurement while retaining information on the whole energy range.

### Data normalisation

Spectroscopic information is collected through the ToF method (Fig. 8)- commonly used in spallation sources - which relies on the measurements of the neutron arrival time on the detector relative to a trigger synchronised to spallation time (*i.e.* when the proton beam hits the target and the neutrons are produced by the spallation reaction). Non-relativistic neutron energies can be derived from the measured ToF *t* as follows:

1 $${E}_{n}=\frac{1}{2}m\frac{{L}^{2}}{{t}^{2}}$$

where *m* is the neutron mass and *L* is the moderator to detector distance. The determination of L has been performed experimentally using the position of main resonances of Gd and Ta foil samples, giving L=23.44 m for this specific experimental campaign.

The available ToF range at the INES beamline is 0–20 ms, which is equivalent in terms of energy to the range 7.2 meV - 11.5 MeV.

Experimentally, the transmission T can be derived by alternating measurements of the transmitted neutron beam with (${C}_{in}$) and without the sample (${C}_{out}$), with background correction for both detected spectra (${B}_{in}$ and ${B}_{out}$) and normalisation to the ratio $M$ between the neutron irradiation currents on the sample and in the absence of the sample:

2 $$T=M\frac{{C}_{in}-{B}_{in}}{{C}_{out}-{B}_{out}}$$

The background has been determined through the black-and-white resonance method^43,44^. The alternation of sample-in and sample-out measurements accounts for the inhomogeneities in the beam from the proton source and variations in the detector efficiency.

Data normalisation and handling are performed through the use of the Mantid software^34,35^.

## Supplementary Information

Supplementary information.

## Data Availability

The datasets generated and analysed during the current study are available from the corresponding author on reasonable request.
