# Supplementary material for: Isotopic imaging with epithermal neutrons at the ISIS Neutron and Muon Source
Source: Sci Rep. 2025 Jun 2;15:19344. doi: 10.1038/s41598-025-04283-y (PMC12130481; doi:10.1038/s41598-025-04283-y)
Supplement: Supplementary file 1 — Supplementary information. [file 41598_2025_4283_MOESM1_ESM.docx]

**Supplementary Material**

**Isotopic imaging with epithermal neutrons at the ISIS Neutron and Muon Source**

**Giulia Marcucci^1,2^ , Antonella Scherillo^2*^, Davide Raspino^2^, Daniela Di Martino ^1^**

^1^ Dipartimento di Fisica “G. Occhialini”, Università degli Studi di Milano-Bicocca and INFN Sezione di Milano-Bicocca, Milan, Italy

^2^ STFC ISIS Neutron and Muon Source, Rutherford Appleton Laboratory, Didcot, UK

* Corresponding author: Antonella.Scherillo@stfc.ac.uk

**Post-irradiation Resonance Selection with the Mantid Project**

The Mantid Project is an open-source software which offers a user-friendly python-based interface (Mantid Workbench) that allows researchers to easily navigate and manipulate neutron scattering (or muon spectroscopy) data acquired at spallation sources, such as the ISIS Neutron and Muon Source. The Mantid Workbench interface is designed to support various scientific analyses, including those related to post-irradiation NRTI data normalization and processing.

Normalised NRTI radiographies can be visualised by selecting the "show instrument" feature, as shown in Figure 1. The 2D map refers to the white beam transmitted through the sample: each pixel in the image stores the relative spectrum in function of the neutron time-of-flights.

**
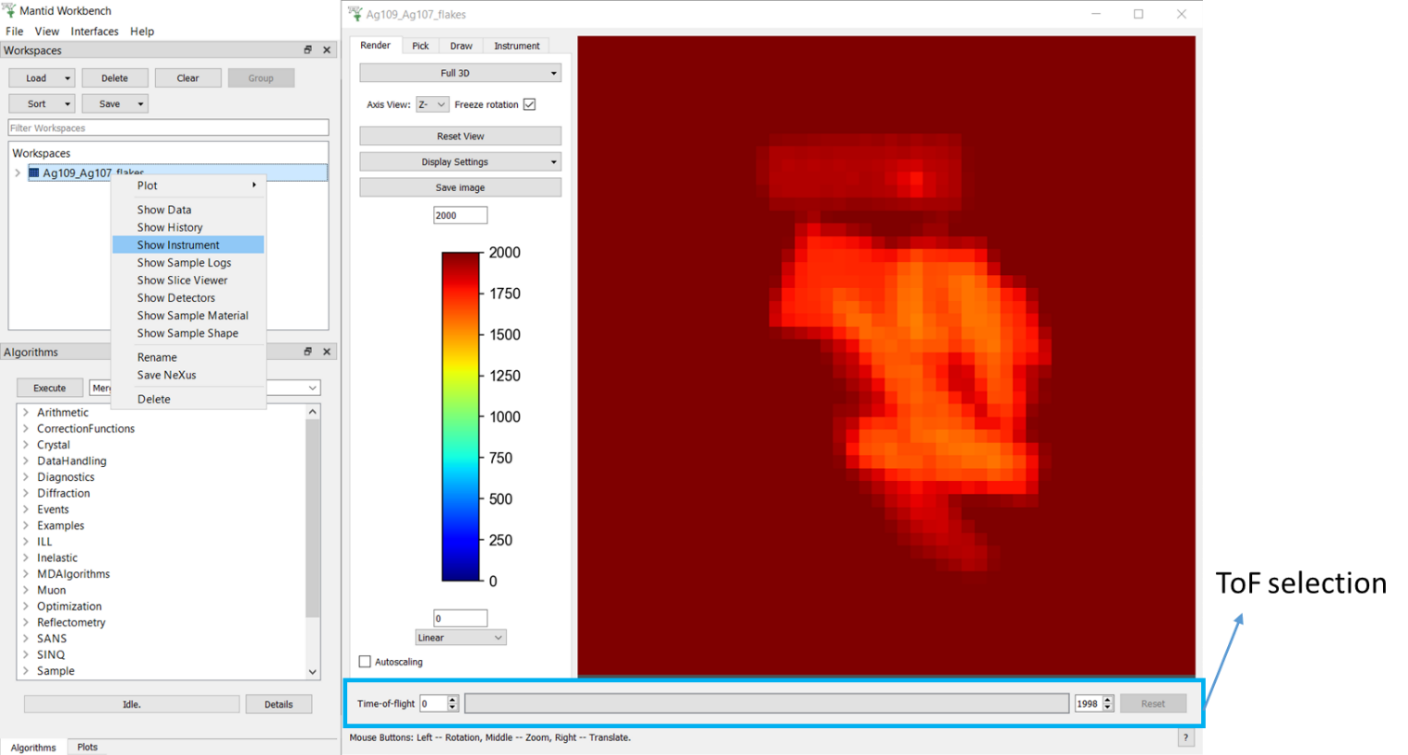
**

**Supplementary Figure 1.** The Mantid Workbench interface with normalised NRTI data visualization through the “show instrument” tool. A separate window opens for the radiography handling. The ToF bar used for resonance selection is highlighted in the blue box.

The blue box in Figure 1 highlights the ToF bar (NRTI data are acquired on INES over the 0-2000 µs range), which is a crucial component for selecting specific time-of-flight ranges and therefore performing the resonance selections. By focusing on particular ToF ranges where resonances occur, it is possible to isolate relevant transmission signal, enhancing in this way the contrast in the 2D map (Figure 2). This selection helps in better interpreting the distribution of the elements and isotopes, allowing for a clearer identification of key features in the sample composition.

Figure 3 shows the "pick" tool, which allows users to select individual pixels of the radiography or to define a Region of Interest (ROI). This feature enhances data analysis by enabling the visualization of spectra in transmission in specific areas of a sample, summing the contributions from the selected pixels. The transmission in function of ToF can be visualized on the left, for quick identification of resonances. More in-depth elemental/isotopic identification can be performed saving the transmission curve in the Mantid interface as a workspace (see user guide available at <https://docs.mantidproject.org/nightly/>).


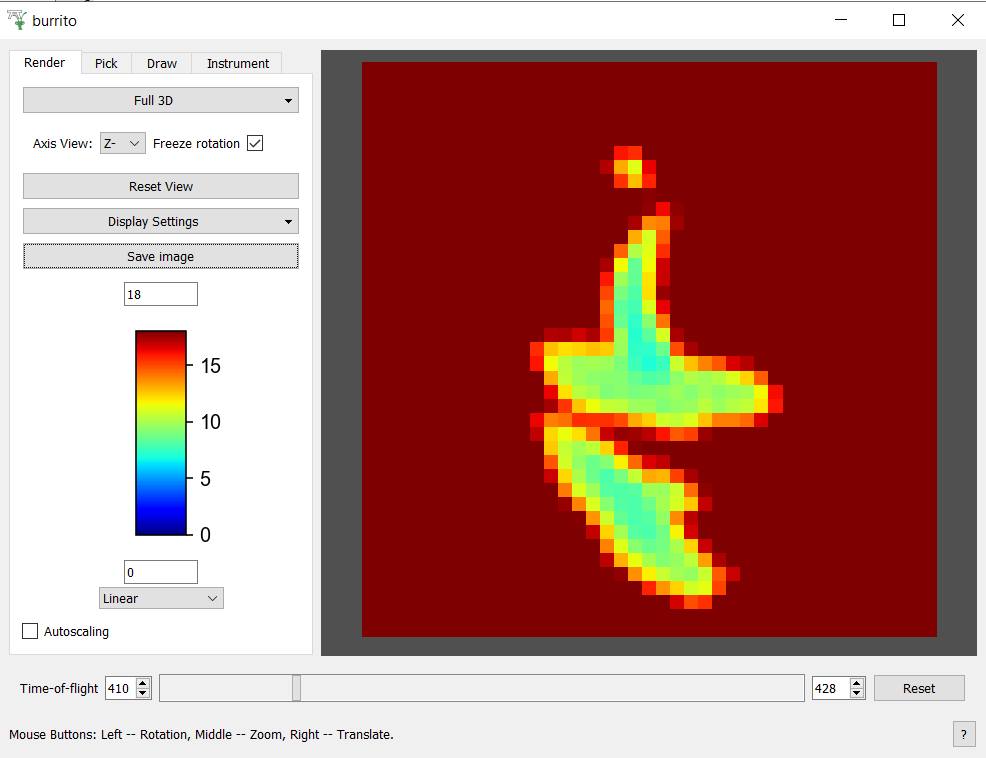


**Supplementary Figure 2.** ToF selection around the resonance of Ag-107 (around 419 µs, i.e. 16.3 eV). The colour bar can be manually adjusted for correct visualization over the selected time range width.

**
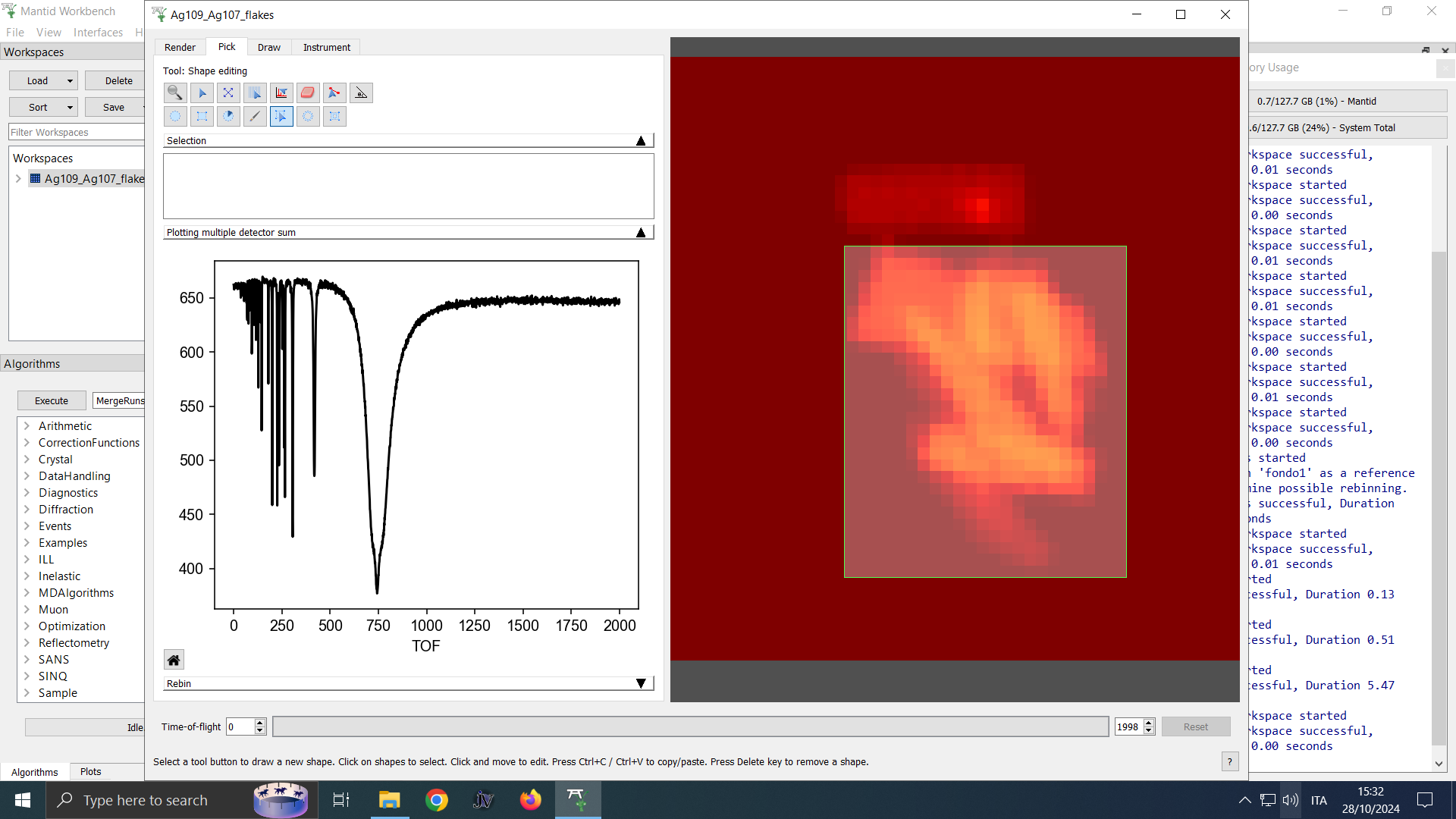
**

**Supplementary Figure 3.** The pick tool enables the selection of single pixels or ROI over the 2D map and the visualization of the corresponding transmission spectrum on the left. It is possible to zoom into the transmission spectrum to more precisely identify the ToF positions of the resonances, enabling isotope recognition and facilitating subsequent resonance selection steps for contrast enhancement.

**Supplementary Figures and Tables**

**
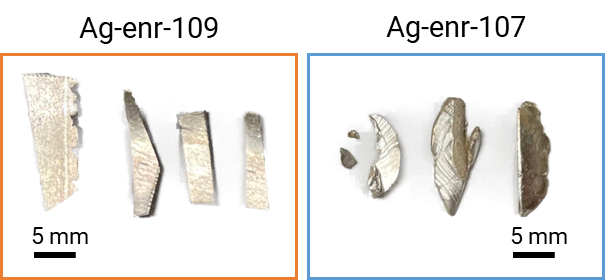
**

**Supplementary Figure 4.** Picture of the “flake” samples enriched with different amounts of Ag-109 and Ag-107.

**Supplementary Table 1.** Isotopic enrichment details of the two types of samples, Ag-enr-107 and Ag-enr-109 and a list of certified chemical impurities. None of these elemental impurities has a particularly intense cross-section to interfere with the isotopic signal characteristic of silver.

| **Isotopic enrichment (%)** | | | |
| --- | --- | --- | --- |
| **Isotope / Flakes type** | **Ag-enr-107** | **Ag-enr-109** | |
| **^107^Ag** | 99.5 +/- 0.1 | 0.3 | |
| **^109^Ag** | 0.3 | 99.7 +/- 0.1 | |
| **Chemical impurities from ICP-MS (ppm)** | | | |
| **Element** | **Ag-enr-107** | **Ag-enr-109** |  |
| Al | 7 | <7 |  |
| Bi | <7 | <7 |  |
| Ca | <10 | <10 |  |
| Cu | 6 | 40 |  |
| Fe | <10 | <10 |  |
| Mg | <10 | <10 |  |
| Na | <10 | <10 |  |
| Si | <30 | <20 |  |
| Sn | 30 | <30 |  |
| Zn | <10 | <10 |  |

**
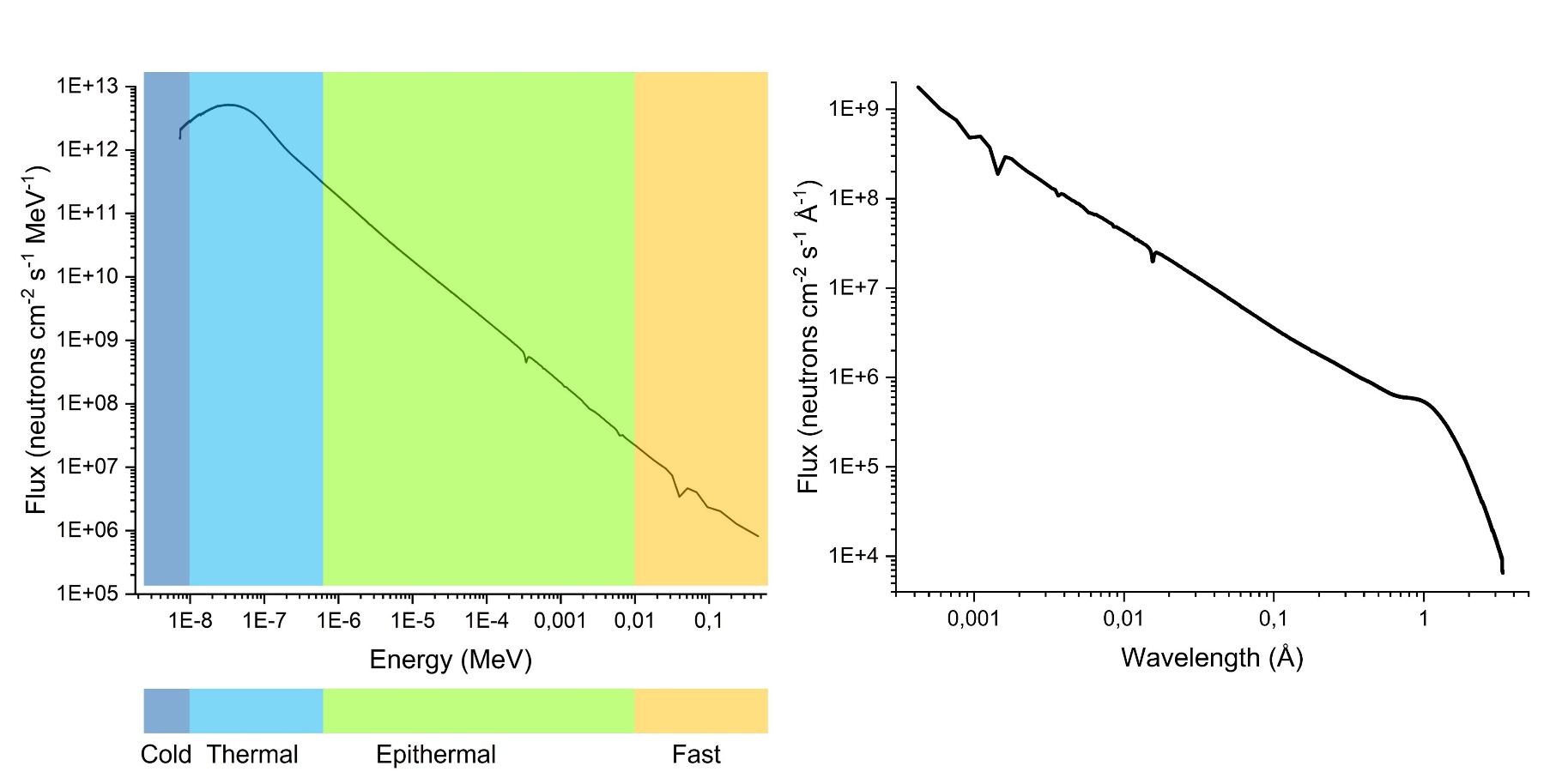
**

**Supplementary Figure 5.** INES neutron flux experimentally measured with the nGEM detector employed for NRTI experiments. Different energy regions are highlighted, from cold to fast ranges.

The INES beamline is characterized by a water-moderated neutron beam (room temperature), but a significant epithermal component (highlighted in green) is also present.
